# Clark County Medical Society

**Published:** 1868-09

**Authors:** F. H. Jennings, J. A. Patton

**Affiliations:** Pres’t.; Sec’y.


### CLARK COUNTY MEDICAL SOCIETY.

This Association met at the court-house, July 1st. President F. H. Jennings in the chair. Fifteen members present.

Dr. F. R. Payne read a paper on Concentrated Organic Remedies. He recommended the use of aconitin, xanthoxylin, fraserin, prunin, hyoscyamin, and atropin, in many diseases. He contended that these concentrated remedies, when pure articles can be obtained, are more convenient for country practitioners, who not only prescribe, but dispense their remedial agents. They are more convenient than fluid extracts.

Dr. Payne then requested the Society to examine a case of displacement of the crystalline lens, in the eye of a little girl, 12 years of age. Dr. Mitchell presented a case of fatty tumor of the chest. Both of these cases were fully discussed, and appropriate treatment recommended.

Dr. R. L. Payne was then made a member of the Society, and read a paper on Puerperal Convulsions, the treatment of which was then fully discussed.

Dr. McCord was in favor of the free use of chloroform, especially after suspended deglutition. He had never witnessed any bad effects from the use of this remedy, and had used it in many diseases, but regarded it as safe as any other remedial agent.

Dr. Jennings was rather afraid of chloroform, but regarded the lancet as the great remedy in this formidable malady, when used at the proper time; but the remedy must be pushed to syncope.

Dr. F. R. Payne contended that there were two forms of puerperal convulsions—centric and eccentric. In the first of these there is congestion or irritation at the nerve centres; but in the second, the irritation is at the periphery of the nerves. He regarded it as very important that the physician should clearly understand these two forms of this disease, as the treatment must, if rational, strictly conform to the true pathology of the disease. We may have convulsions in a pale, cachectic woman, caused by improper or indigestible food, and in such cases the lancet would be very improper; but in all plethoric, short-necked, full-blooded patients, he regarded the lancet as the sheet-anchor and only hope.

Dr. McCord agreed with Dr. Payne, in his remarks, and regarded this as one of the most formidable diseases of the lying-in chamber; and every physician should thoroughly comprehend its true pathological character.

Dr. F. R. Payne was of the opinion that this disease might be prevented, in a great majority of cases, by proper remedies. He invariably recommended in all primapara cases of full habit, the free use of saline purgatives for two or three months previous to confinement, and has no fear of puerperal convulsions.

Dr. McCord verbally reported a case, where medicines could not be administered internally, and the patient was pronounced beyond the reach of remedies; but by the continued and cautious use of chloroform she recovered.

Dr. Price then read a paper on Indigestion, which was fully discussed and referred to the Publishing Committee. He gave subnit. bismuth, with antacids, zingiberis, and sodæ. This paper entered fully into the etiology and pathology of the disease, and evinced thorough research on the part of the author.

Dr. McCloud read a paper on Hemorrhage, but, at his own request, he was permitted to retain the manuscript, to be completed at the next meeting of the Society.

Dr. Payne then read a paper on Abortion, which was freely discussed by nearly all the members; after which, Dr. Mitchell offered the following resolution, which was unanimously adopted:

*Resolved*, That a committee of one in each township be appointed, to report, at our next meeting, all cases of abortion in their respective townships, and ascertain, if possible, whether they were *procured* or *accidental*.

In the discussion of this resolution, it was conceded that criminal abortions were fearfully frequent, and that the crime, as a general rule, was committed by irregular practitioners, druggists, and pretended female accoucheurs; and that it was the duty of all true physicians to aid the officers of the law in prosecuting such offenders.

Dr. Steelsmith was proposed as a member of this Society; but, he being a stranger to all present, his case was referred to the Board of Censors.

A case of idiocy was brought before the Society, for examination, by request of the County Board of Supervisors, and the following resolution was unanimously passed, after examination and inquiry in regard to the case:

*Resolved*, That, having examined John White, one of the County poor, we believe him to be idiotic, yet possessed of slight intelligence, and may be able to learn some useful business if properly instructed.

A long discussion was then had on the duties of the physician, medical ethics, etc.

This meeting was one of unusual interest, and many important subjects were before the Society; many gems of practical experience were produced for mutual benefit; and, we are happy to say, the best of feeling prevailed. We are confident that since the organization of this Society there has been a better feeling prevailing among the physicians of Clark County, and that the people are becoming satisfied that we do not meet for the purpose of combination, thus enabling us to charge "*unreasonable bills*," but that the object is to secure mutual improvement, and thus, by consultation and discussion, better qualify ourselves to successfully combat the diseases of this locality.

F. H. JENNINGS, *Pres't.*

J. A. PATTON, *Sec'y.*

